# Recurrent melanoma presenting as a rash: a case report

**DOI:** 10.1093/skinhd/vzaf073

**Published:** 2025-10-01

**Authors:** Annabelle Huntsman, Katherine Gillis, Anneli R Bowen, Dekker C Deacon

**Affiliations:** School of Medicine, University of Utah, Salt Lake City, UT, USA; School of Medicine, University of Utah, Salt Lake City, UT, USA; Department of Dermatology, University of Utah, Salt Lake City, UT, USA; Department of Dermatology, University of Utah, Salt Lake City, UT, USA; Huntsman Cancer Institute, Salt Lake City, UT, USA

## Abstract

Melanoma is the most aggressive form of common skin cancer and recurrence, while rare, typically presents as pigmented papules or nodules near the primary site. Here, we describe a case of a woman with stage IIIC melanoma undergoing intralesional talimogene laherparepvec (T-VEC) therapy who developed a spreading erythematous rash on her left leg, accompanied by fatigue and leg swelling. Skin biopsy revealed recurrent melanoma, with SOX10 and Melan-A positivity, and imaging showed features concerning for multifocal disease recurrence in the left popliteal fossa. This case highlights an unusual presentation of melanoma recurrence and underscores the importance of biopsy in ­evaluating new skin findings in patients with a history of melanoma.

## Teaser

Melanoma often presents as a brown to black pigmented lesion with changes in size, shape or colour. Our study presents a unique case of recurrent melanoma presenting as a rash following a T-VEC injection.

What is already known about this topic?

- Melanoma usually presents as a brown-to-black pigmented lesion with noticeable changes in size, shape or colour.
- When melanoma recurs, it often presents as a new pigmented lesion or a pre-existing lesion, typically near the original site.
- Few published cases of documented recurrent melanoma presented as a rash; the only related case reported was recurrent melanoma masquerading as a zosteriform rash.

What does this study add?

- This study highlights a unique case where the patient’s recurrent melanoma presented as a changing rash, a presentation not previously reported.
- Our clinical report includes a high-quality clinical photograph from the patient presentation, as well as the histopathology results, which confirmed the diagnosis of recurrent melanoma, emphasizing the importance of biopsy in unusual clinical presentations.

Cutaneous melanoma is a highly malignant tumour with overall recurrence rates in patients with stage I–III disease reported as high as 8.8%.^1,2^ Recurrence may occur via lymphatic or haematogenous spread and may present as local, in-transit, regional lymph node or distant metastases. The risk and timing of recurrence are closely tied to the initial tumour stage, with stage III disease most often recurring within the first 2–3 years after diagnosis.^3^ Notably, recurrence is most often detected through patient self-report of new or changing symptoms, highlighting the importance of patient education and vigilant follow-up.^3^ When melanoma recurs, it often presents as a new pigmented lesion or a pre-existing lesion near the original site with noticeable changes in size, shape and colour.

This case describes an atypical presentation of metastatic melanoma recurrence in a patient undergoing talimogene laherparepvec (T-VEC) therapy, initially appearing as a spreading erythematous rash. It underscores the importance of histological evaluation to differentiate treatment-related skin reactions from recurrence. The case also highlights the need for ongoing patient vigilance and provider awareness during follow-up, as early detection allows for more timely and effective treatment.

## Case report

A 59-year-old woman was referred to dermatology with a rash on her lower left calf. The patient reported that the rash on her left lower calf had been present for 1 week, and had increased in size and slowly progressed to her knee and upper leg. The rash was accompanied by fatigue, itchy eyes and leg swelling.

The patient had a personal history of stage IIIC melanoma on her left posterior calf, for which she was undergoing intralesional treatment with T-VEC. Prior to the onset of her rash, she had received two injections of 0.5 mL T-VEC, both delivered into a single lesion at the site of her primary tumour. The most recent injection was administered 4 days before the rash developed.

The patient had previously completed nine cycles of pembrolizumab, which was discontinued due to ­immunotherapy-­induced arthritis, and a course of BRAF/MEK inhibitors, which was stopped due to tinnitus. Her ­clinical course was notable for metastatic spread to the sentinel lymph nodes of the left groin, without evidence of bone, brain, lung or other visceral involvement. Her family history was significant for melanoma in her father.

## Clinical findings

**Figure 1:**
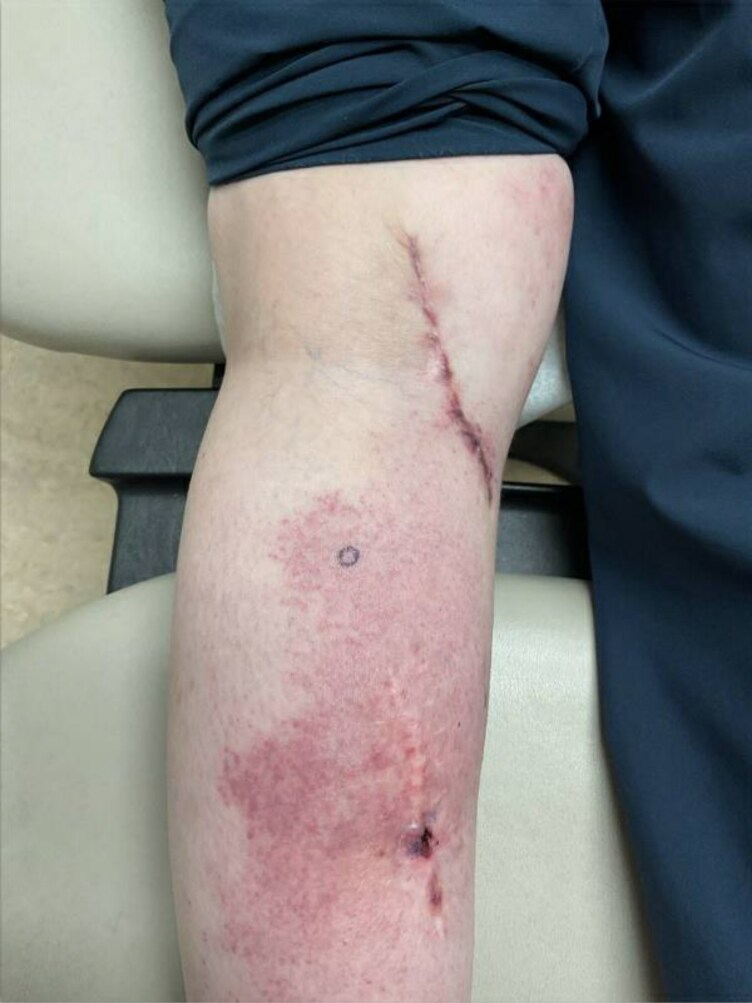
Melanoma recurrence presenting as a rash consisting of pink-to-red macules coalescing into patches and thin plaques. The biopsy site is circled in purple. Linear scars on calf and popliteal fossa represent site of primary melanoma and oligometastatic disease resections respectively. Talimogene laherparepvec (T-VEC) was previously administered to the site of primary disease on patient’s left medial calf.

On physical examination, light pink-to-red macules and papules coalescing into patches and thin plaques on the left calf and popliteal fossa and extending to the left shin were observed (Figure 1). On the left calf there was a linear scar with central crusting, as well as a linear scar with purple discolouration and blanching in the left popliteal fossa, consistent with prior surgeries, including resection of the primary melanoma and subsequent oligometastatic disease in the popliteal fossa.

## Timeline

### Diagnostic assessment

Prior to presentation for rash, the patient had been diagnosed with superficial spreading melanoma on the left posterior calf. Imaging, including computed tomography (CT) of the chest and abdomen/pelvis, as well as brain magnetic resonance imaging (MRI), showed no evidence of distant disease. A year before rash onset, the patient exhibited local recurrence in the skin of the left popliteal fossa, proximal to the site of primary resection, with in-transit metastatic melanoma confirmed by fine-needle aspiration.

**Figure 2:**
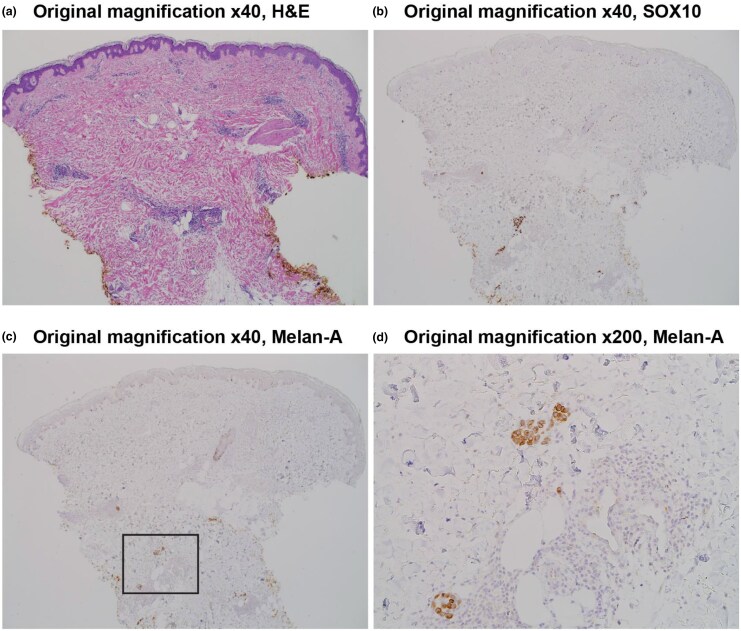
(a) Haematoxylin and eosin (H&E) stain demonstrating perivascular focally dense lymphocytic infiltrates. (b) SOX10 and (c, d) Melan-A immunostaining reveal micrometastatic melanoma deposits.

**Figure 3:**
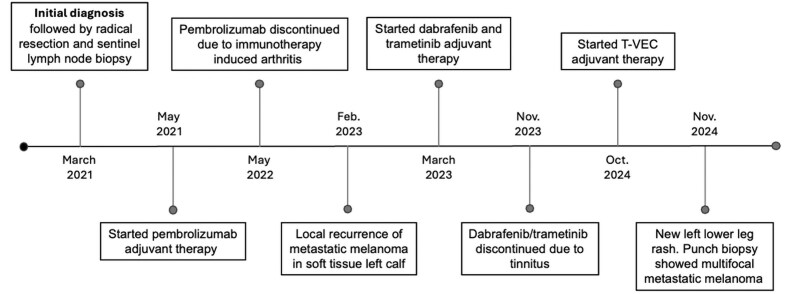
Full timeline of the patient’s diagnosis, work-up and treatment.

The patient presented with a spreading erythematous rash on the left lower extremity near the site of her previous primary melanoma, 4 days after receiving her second T-VEC injection. On physical examination, the rash consisted of scattered erythematous patches and plaques extending proximally from the prior tumour site. Given the timing and appearance, the initial clinical impression favoured a local inflammatory or allergic reaction to T-VEC, an oncolytic virus known to cause injection-site rashes.^4^ The differential diagnosis also included cutaneous small vessel vasculitis^5^ and postsurgical stasis dermatitis,^6^ which can present with similar clinical findings. To clarify the aetiology, a punch biopsy was performed. Histopathological evaluation revealed numerous collections of atypical melanocytes, consistent with metastatic melanoma. Immunohistochemical staining was positive for melanocyte markers, SOX10 and Melan-A, further supporting this diagnosis (Figure 2). A timeline of the patient’s diagnosis, work-up and treatment is provided (Figure 3).

## Therapeutic intervention

Prior to presentation for the rash, the patient had completed nine cycles of pembrolizumab (400 mg intravenously every 6 weeks), which was discontinued due to ­immunotherapy-induced arthritis. After exhibiting local recurrence, she underwent an 8-month course of BRAF/MEK inhibition with dabrafenib (75 mg twice daily) and trametinib (2 mg once daily).

Following rash presentation, the patient underwent further imaging, including brain MRI and whole-body positron emission tomography (PET)/CT, which revealed increased uptake in the left popliteal fossa. After meeting with ­medical-oncology and discussing tumour-infiltrating lymphocyte therapy and other available options, the patient opted to continue T-VEC injections every 2 weeks. T-VEC was favoured over immune checkpoint inhibitors and other systemic therapy due to her previous reaction, leading to ­immunotherapy-induced arthritis.

## Follow-up and outcomes

The patient’s rash resolved approximately 1 month after presentation. She subsequently completed seven additional T-VEC injections without notable side effects and demonstrated a strong therapeutic response. Six months after presentation, PET/CT showed no evidence of residual or metastatic disease.

## Discussion

Malignant melanoma is a type of skin cancer that arises from melanocytes and is recognized as the most aggressive form of the common cutaneous neoplasms.^7^ Risk factors for melanoma, as highlighted in the patient’s history, include lifetime ultraviolet exposure and a first-degree family history of melanoma.

Cutaneous melanoma recurrences typically present as dark-brown or black papules and nodules adjacent to primary disease sites, although recurrence as amelanotic pink papules and nodules can occur.^1^ Even rarer is the presentation of melanoma as a rash, which has been reported previously with zosteriform morphology,^8^ emphasizing the importance of diagnostic scrutiny and histopathological analysis of unusual presentations to identify melanoma recurrence as soon as possible.

Clinically, melanoma typically appears as a dark-brown-to-black papule that is either enlarging, changing in colour or border, or becoming asymmetric. Patients are encouraged to monitor their ‘ABCs’ (asymmetry, borders, colour) for any suspicious or changing features. The gold standard for diagnosing melanoma remains a biopsy of the lesion with an associated histopathological analysis.^9^ In this case, the diagnosis was confirmed with histopathological analysis, which revealed numerous collections of melanocytes, that stained positive for SOX10 and Melan-A, both common markers for melanoma. Current treatment approaches for melanoma include surgical resection of the lesion, with adjuvant or neoadjuvant systemic treatment for advanced-stage disease.^10^

The pattern of melanoma recurrence in this patient, presenting as a rash, is unusual, and highlights the diagnostic value of skin biopsy in distinguishing treatment-related effects from true disease progression. This study highlights a unique case where the patient’s recurrent melanoma presented as a changing rash, a presentation not previously reported.

## Data Availability

All data are presented in the article.
